# Analysis of anthropogenic impact on the environment, measures to reduce it, and waste management

**DOI:** 10.3389/fbioe.2023.1114422

**Published:** 2023-03-28

**Authors:** Nadezhda A. Eremeeva, Olga A. Savoskina, Liudmila M. Poddymkina, Khamzat A. Abdulmazhidov, Abdurachman G. Gamidov

**Affiliations:** Russian State Agrarian University—Moscow Timiryazev Agricultural Academy, Moscow, Russia

**Keywords:** biotechnology, waste, processing, air, water and soil pollution, human waste

## 1 Introduction

The release of organic waste annually amounts to millions of tons of substances. Waste is used as a raw material for fertilizers and microbiological technologies. Anthropogenic pressure increases the volume of waste, which is important for large cities. The release of sewage sludge is expressed in millions of tons. It is disposed of in landfills. Waste is generated as a result of the use of natural resources; it serves as a source of raw materials and secondary raw materials. Waste from the food and processing industries can be recycled. Protein raw materials are used to produce supplements, dietetic treatments, and growing mediums. Microorganisms can decompose organic matter. Ecobiotechnologies use this ability for microbiological synthesis. The main methods of environmentally friendly waste processing are composting and vermicomposting, methane digestion, and aerobic stabilization. Composting is of low cost. Vermicomposting allows obtaining biohumus, which is technologically advanced in use. Liquid fractions of manure, droppings, and manure containing urea are dangerous for the environment. The pollution of nature is a catalyst for ecological catastrophe. Thus, a comprehensive analysis of environmental pollution is needed.

Microorganisms and their enzymes destruct organics, which makes it possible to obtain biofertilizers with a high agronomic effect (Zainutdinov et al., 2010; Bogatova et al., 2012; Guber et al., 2013). Composting in animal husbandry has low capital and operating costs. Vermicomposting ensures the production of biohumus with high moisture capacity, flowability, which has associations of microorganisms useful for plants and soil (Titova, 2004). The main factor in the violation of the integrity and structure of natural communities, changes in the properties of the natural environment is human activity (Zinina et al., 2010; Zainutdinov et al., 2011; Zinina et al., 2013). The growth in the amount of waste gives rise to the need to search for new and optimized known methods for the neutralization and disposal of substances harmful to nature and humans. The following are widely used: combustion, pressing, aerobic fermentation (Okuskhanova et al., 2014). Specific methods have advantages and disadvantages and are applied depending on the expediency of application in local conditions.

Traditionally, wastes are recycled naturally, with the participation of relevant microorganisms (Tarasova et al., 2013; Zavrazhnov et al., 2014; Ngozi and Arihilam, 2019). Wastewater treatment plants perform such operations as (1) the removal of solid particles, which are either disposed of in a landfill or sent to a bioreactor, (2) the destruction of dissolved organic matter using natural aerobic microorganisms, (3) the realization of chemical precipitation and separation of phosphorus and nitrogen, (4) and for sludge processing, the application of anaerobic digestion to reduce the volume of sludge and the number of pathogenic microorganisms, and eliminate odor (Pakhnenko, 2016). The resulting methane is a valuable organic fuel. The same processes are used in the chemical, food, and pulp and paper industries (Tuna, 2011; Hammer et al., 2013; Cook, 2016). Organic waste processing technologies include (1) composting, (2) dry extrusion, (3) anaerobic bioconversion, (4) plasma gasification, (4) thermal depolymerization, (5) vermiculture, (6) biotechnologies (the application of larvae, flies, annelids, and biohumus), and (7) vacuum drying.

Green plants are also used in wastewater, soil, and atmospheric air purification (phytoremediation method), which is one of the directions of bioremediation. Anthropogenic pressure on ecosystems is growing, but sustainability has limits; the current problem is to develop biological methods for cleaning the environment. These methods are based on natural mechanisms. One of these methods is phytoremediation - the purification of the environment from pollutants with the help of living plants that can absorb heavy metals and their toxic compounds (Mironov, 2018). Phytoextraction uses plants to extract pollutants from the environment by transporting metals into plant tissues through roots. This method is used to remediate Pb, radionuclides with Cr, As and Hg, Ni, Cu. Plants are extracted from soil and water with As, Cd, Cu, Hg, Se, and Pb. The plant mass is collected and burned; the resulting ash is buried or used as a secondary raw material. Phytoremediation is used to clean soil from petroleum hydrocarbons, pesticides, chlorinated solvents, and industrial wastes and to remove contaminants from surface aquifers. Methods resulting in volatilization are used to neutralize volatile pollutants (phytovolatilization).

The main directions of biotechnological waste processing are based on the degradation of toxic waste; the return of carbon, nitrogen, phosphorus, and sulfur into the cycle of substances; as well as obtaining organic fuel (Hoffman et al., 2015). The aim of the study was to analyze the use of contemporary biotechnologies for processing organic waste and reducing the anthropogenic impact on the environment. Our developed system for analysis provides for obtaining information on the pollution sources, the impact of non-decontaminated waste on the environment, and assessing the reaction mechanism of natural entities to anthropogenic substances. We also created a system for assessing critical levels in the biosphere, dosing the load, environmental control, and regulation of biosphere environments.

The research novelty lies in the fact that we propose a system of a comprehensive analysis of the state of the environment, which provides for obtaining objective data on the sources of environmental pollution, the nature of the impact of pollutants on the natural environment, people, and animal and vegetal life, identifying the mechanism of the response of natural objects to the impact of harmful substances on the animal world and nature. It is possible to identify critical links in the biosphere, normalize anthropogenic impact, and environmental-economic regulation of the natural environment using the proposed method.

## 2 Monitoring and reducing the level of environmental pollution to assess critical levels in the biosphere and the dosage of anthropogenic load

Global environmental problems require significant efforts of the entire international community. Humankind has destroyed and continues to destroy up to 70% of natural ecosystems on the planet in the 20th century, which are capable of retransforming waste products. The permissible anthropogenic impact on the biosphere has now been exceeded several times. Thousands of tons of substances of various nature are thrown into the natural environment, which are not amenable to natural processing. The most urgent environmental problem is protection from pollution, which includes the protection of the atmosphere, hydrosphere, and lithosphere. Table 1 presents biotechnological methods for neutralizing and processing organic waste with indicators of quantitative costs in world prices.

**TABLE 1:** Biotechnological methods for the neutralization or processing of organic waste.

Type of organic waste	Type of biorefinery	The number of costs for technological solutions for recycling, according to world prices/thousand $
Manure and droppings	Vermicomposting, production of organomineral fertilizers, processing into feed	3,120.0
Crop waste	Methane digestion in anaerobic bioreactors, production of protein and biofuel	12,248.0
Waste with carbohydrates, fats, and proteins	Obtaining food products, feed protein, and enzymatic processing	9,240.0
Solid protein- and fat-containing waste	Obtaining food additives, BAS, products of microbiological processing, organomineral fertilizers	10,000.0
Sediments and sludge from sewage treatment plants	Methane digestion in septic tanks, composting, aerobic stabilization, aging in sludge beds	12,600.0
Organics of municipal solid waste	Composting, burial at bioreactor landfills, methane digestion	29,000.0

Microorganisms can decompose organic substances and transform polymers. Ecobiotechnologies use this ability during microbiological synthesis in waste processing. The main methods of deep, environmentally friendly waste processing are composting and vermicomposting (Merzlaya, 2001; Titova, 2004), methane digestion, and aerobic stabilization for biogas production. At the same time, composting has the lowest costs. Processing of organic waste by vermicomposting makes it possible to obtain vermicompost and biomass of earthworms. Biohumus is technological in use; it normalizes the development of microbial associations and ensures the suppression of pathogens.

**FIGURE 1:**
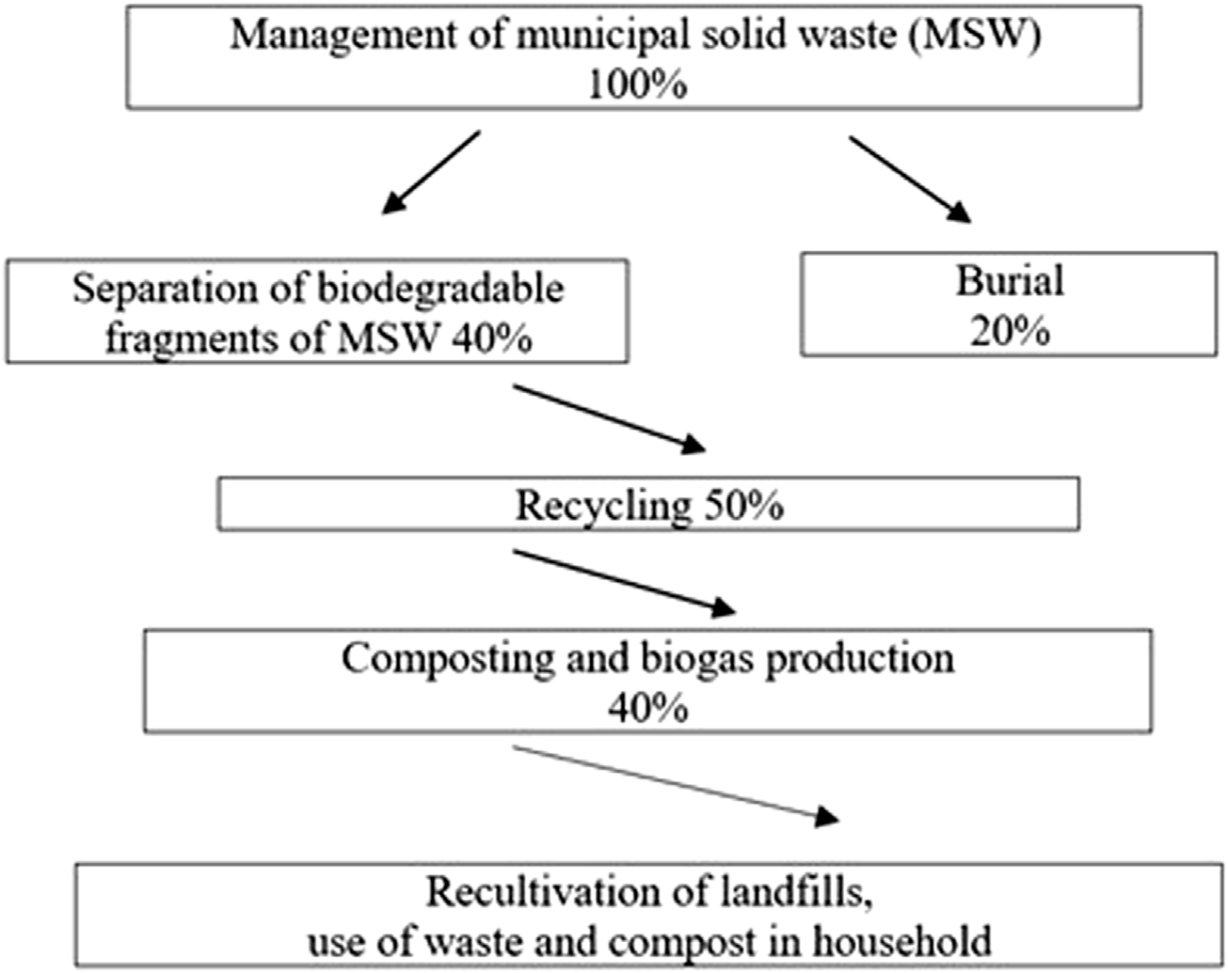
Block diagram of industrial waste processing.

Only a small part of cattle manure and bird droppings is used as fertilizer. The rest of the agricultural production waste accumulates and negatively affects the environment. Liquid fractions of manure, droppings, and manure sewage contain urea, phenols, pathogens, drugs, and other environmentally harmful substances (Guber et al., 2013; Zavrazhnov et al., 2014). Most of the dry matter is disposed of in landfills, and only a part is used as fertilizer; they are buried. Half of the municipal solid waste is the organic part used as raw materials to manufacture fertilizers in agriculture. The block diagram below depicts the complex path of household waste - from managing the recycling process to remediation and use (Figure 1) (European Comission, 2019).

The most common methods of waste processing and reactivation are thermal (burning, gasification, pyrolysis), which produce carbon dioxide, water vapor, nitrogen and sulfur oxides, carbon monoxide, dioxins. Pyrolysis is used to produce activated carbon from wood. The main negative factor of thermal utilization is the release of dioxins into the environment (Mironov, 2018). Organic waste has a diverse negative impact on the environment. Due to industrial, agricultural, and domestic human activities, the physical, chemical, and biological properties of the natural environment change. These processes are used in agriculture, with bioreactors being built to process waste. New bioreactors use bacteria and sunlight to convert CO_2_ into acetate (Markov et al., 2013).

Land waters have atmospheric nutrition, and their composition depends on the state of the atmosphere. The polluted atmosphere is characterized by acid precipitation, which leaches macro- and microelements, humus from the soil, disrupts plant photosynthesis, slows down their growth, and causes the death of plants sensitive to pollution. All these factors lead to a decline in soil fertility and, as a result, to the disappearance of forests (Lev, 2016).

Aerobic wastewater treatment is the most extensive use of microorganisms in biotechnology. It is promising for the economical production of gaseous fuels at moderate temperatures (30°C–35 °C). Different countries use different types of waste to produce energy and by-products. Brazil, Australia, and New Zealand use specialty crops for fuel. Similar projects are being discussed in Finland, Sweden, and Ireland. In England, work on energy bioconversion is carried out under the Solar Energy Program, and biological energy projects are heavily financed there.

The 20th and early 21st centuries show an increase in environmental accidents and catastrophes (Tuna, 2011; Hammer et al., 2013; Ngozi and Arihilam, 2019). Pollution of nature is a catalyst for ecological catastrophe, with the following sources of pollution: industry, transport, energy and agricultural complexes, fertilizers, pest control products, emissions of solid and liquid wastes of various nature. Physical, chemical, and biochemical processes of unknown nature and unpredictable consequences occur at industrial and domestic waste landfills, which lead to the synthesis of toxic compounds in different physical states. Household waste is favorable for the reproduction of various types of insects, birds, rodents, microorganisms, which can have a negative biogenic effect on humans and animals. Birds and insects can be vector carriers of pathogenic bacteria and viruses. Unfortunately, there are currently no ecosystems on Earth that are not affected by human irreversible processes.

## 3 Conclusion

Chemical and biochemical enterprises are carriers of the potential danger of accidental releases of toxic substances into the atmosphere and microbes that can cause epidemics among people and animals. The ecological situation on the planet is unfavorable due to the insufficient use of the potential of nature by scientific and technological progress and the irrational exploitation of natural resources, increasing the rate of pollution of the biosphere. Humanity has novel methods and approaches to normalize the current situation by processing industrial organic waste. Normalization provides for reducing the degree of pollution of the natural environment; rationalization of exploitation of natural resources; reactivation of non-renewable resources; use of environmentally friendly biotechnologies. Environmentally friendly biotechnologies are the main and promising way to combat pollution of the natural environment, atmosphere and hydrosphere, and the fertile soil layer. A comprehensive analysis of the state of the natural environment is much needed in scholarship and practice, considering promising ways of using biotechnology. The analysis provides a reliable idea of the sources of environmental pollution, the nature of the impact of non-decontaminated waste on the environment and wildlife, the establishment of a mechanism for the reaction of natural subjects to anthropogenic substances and energy fields.
